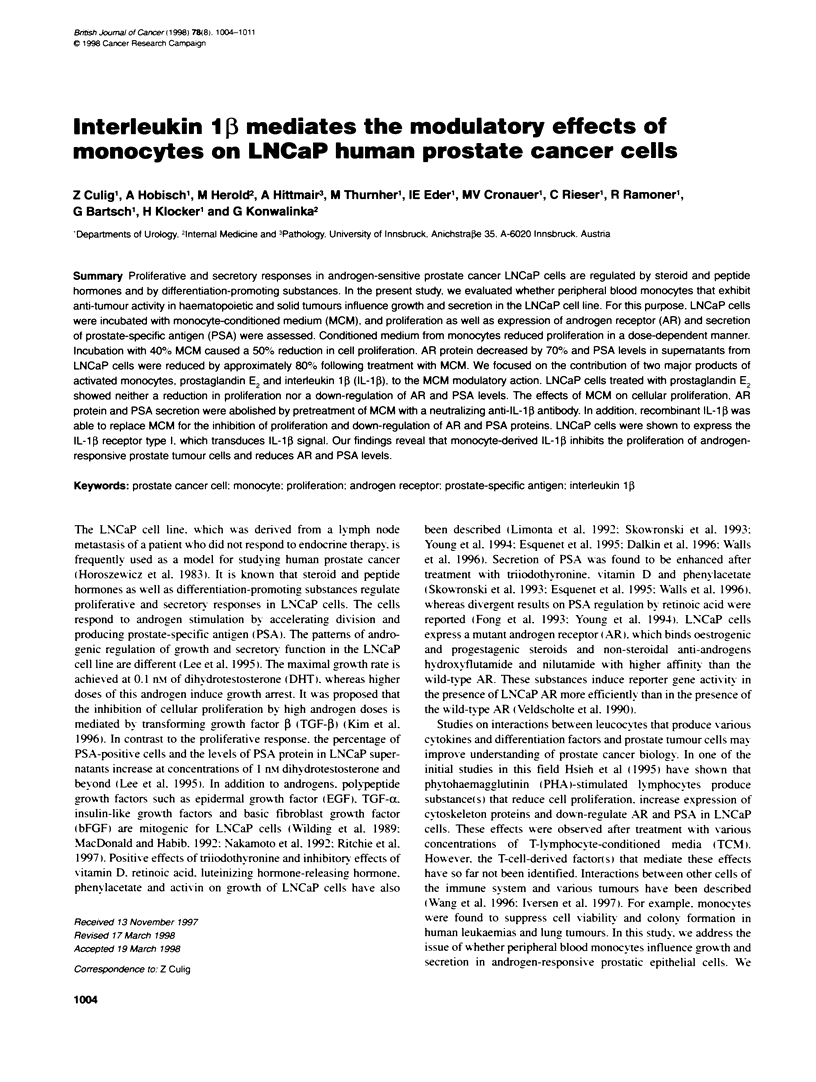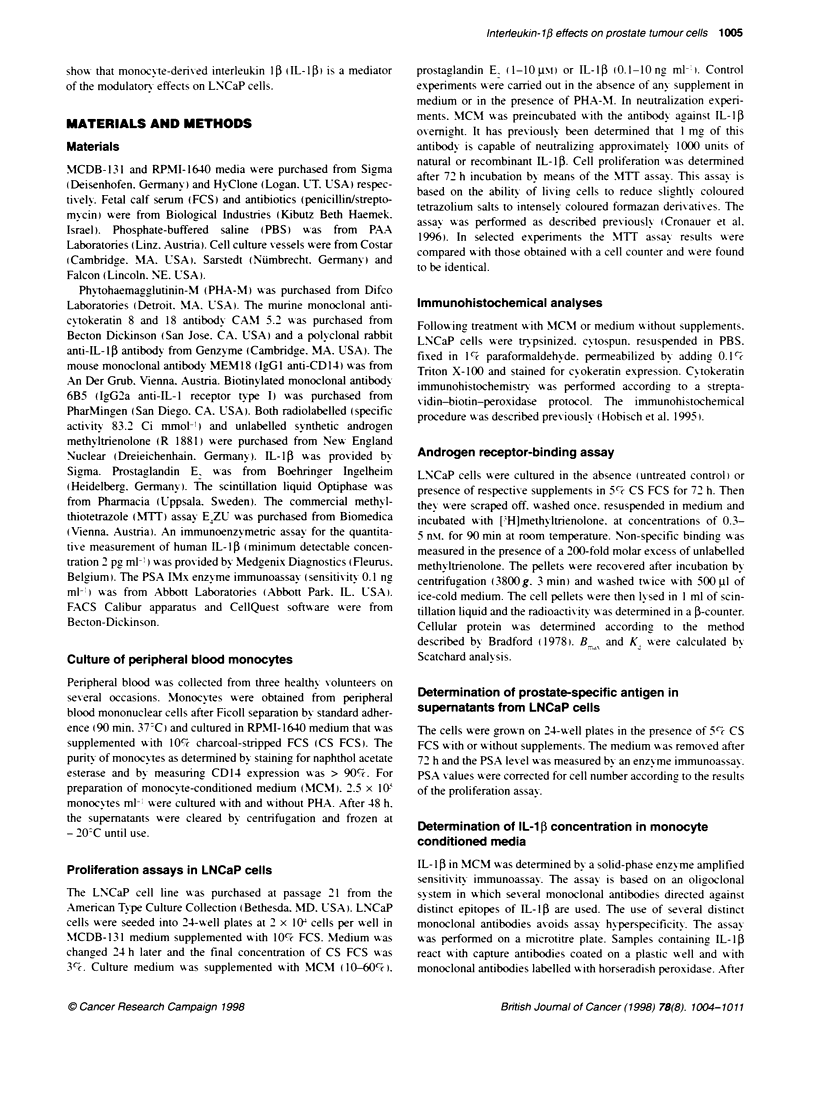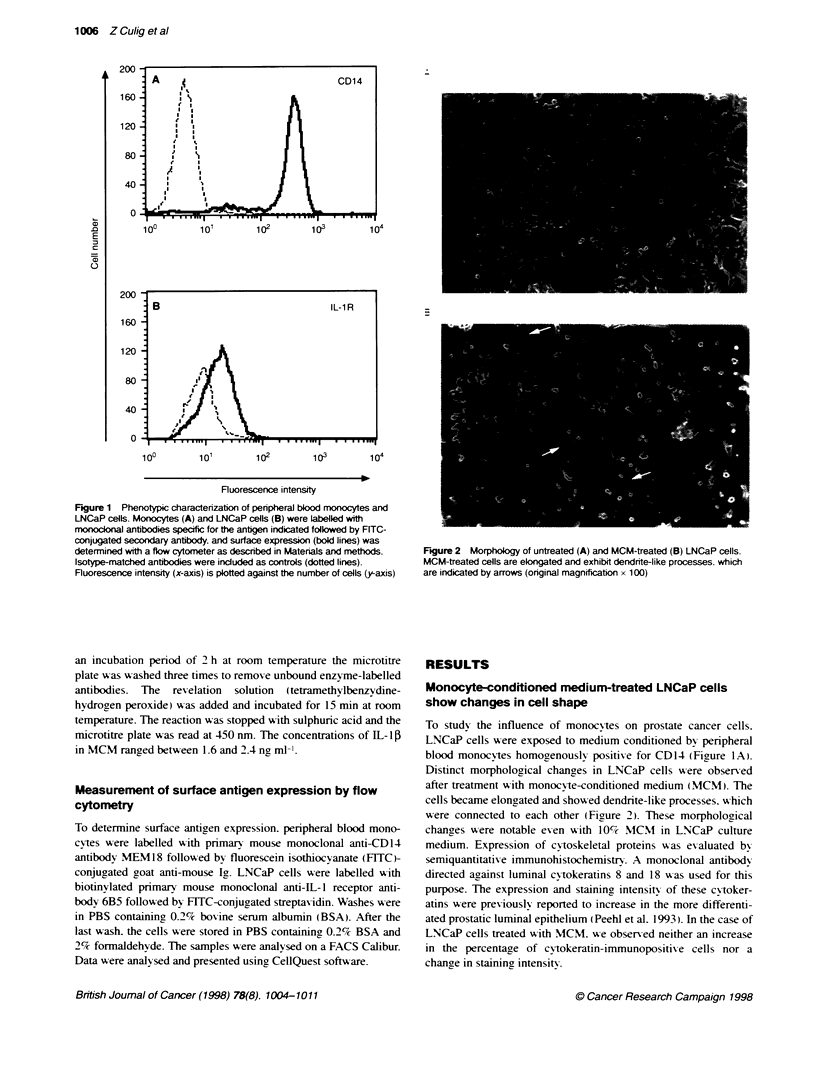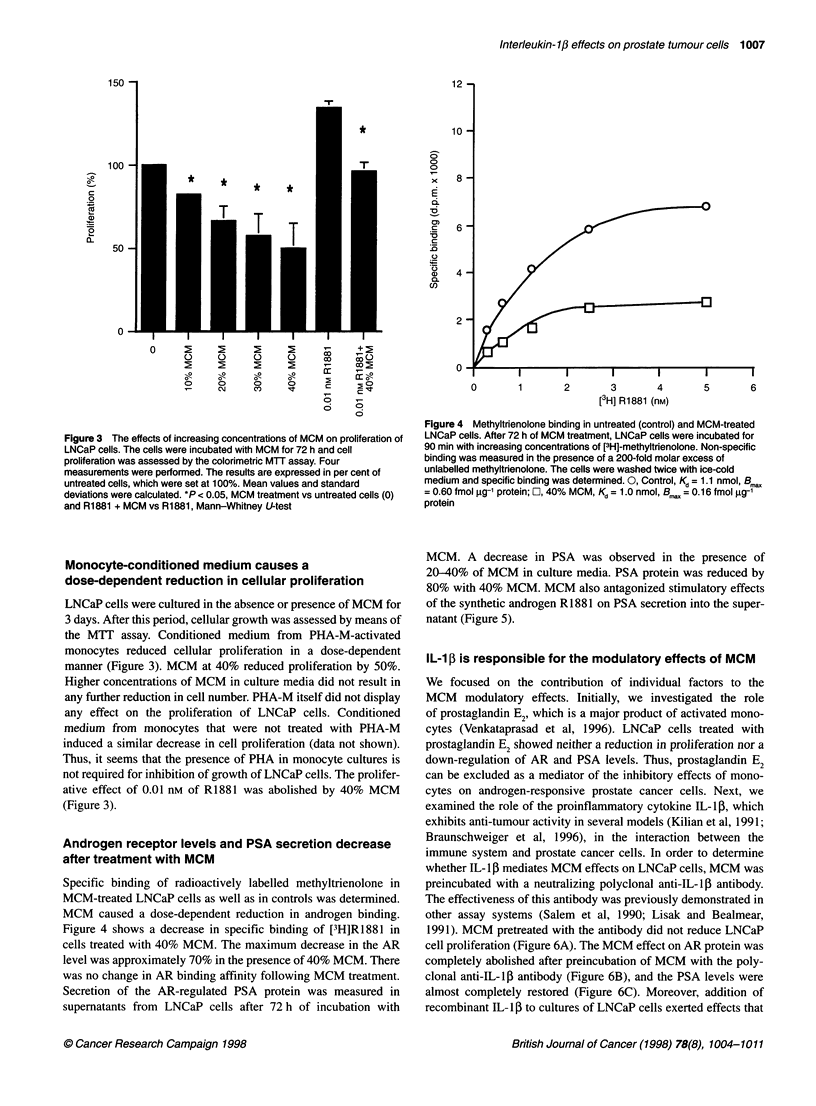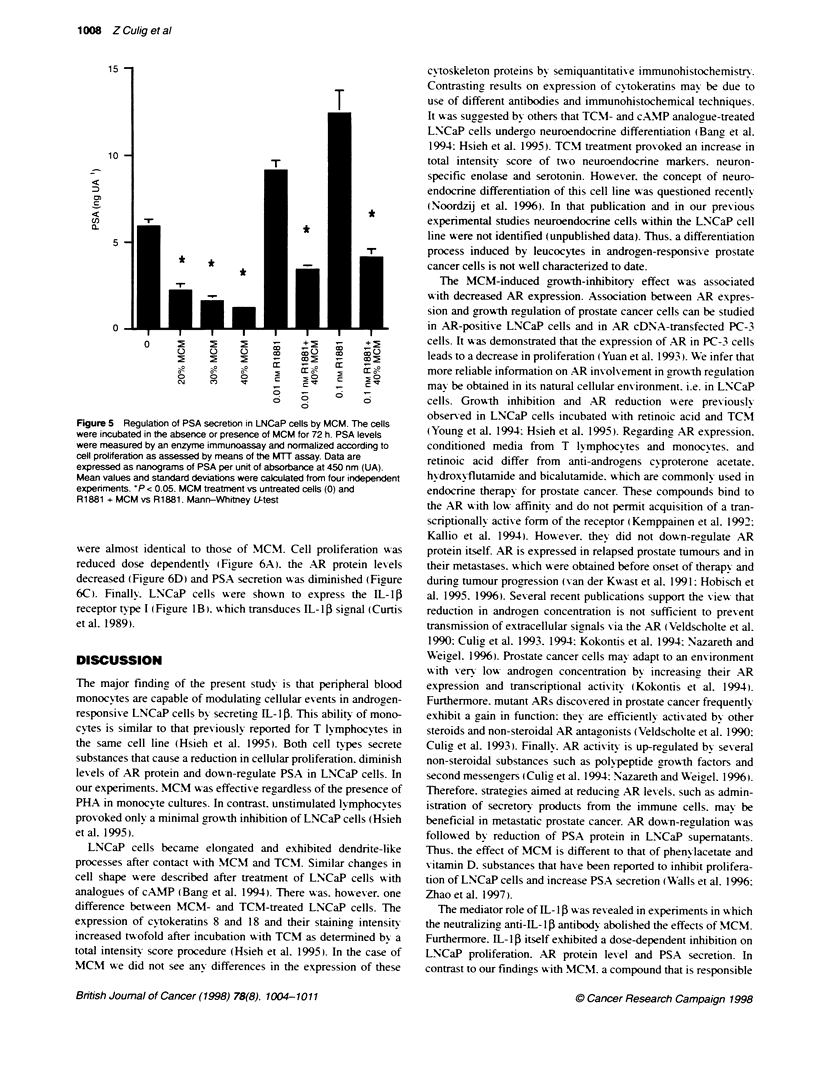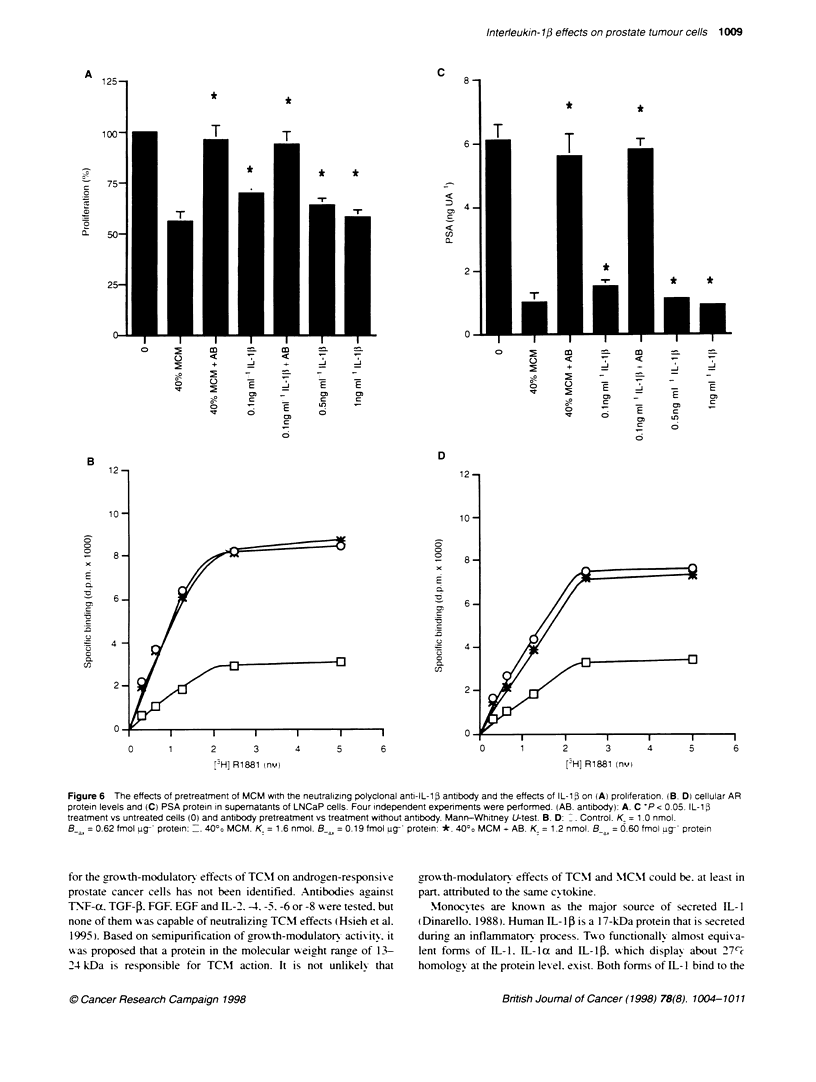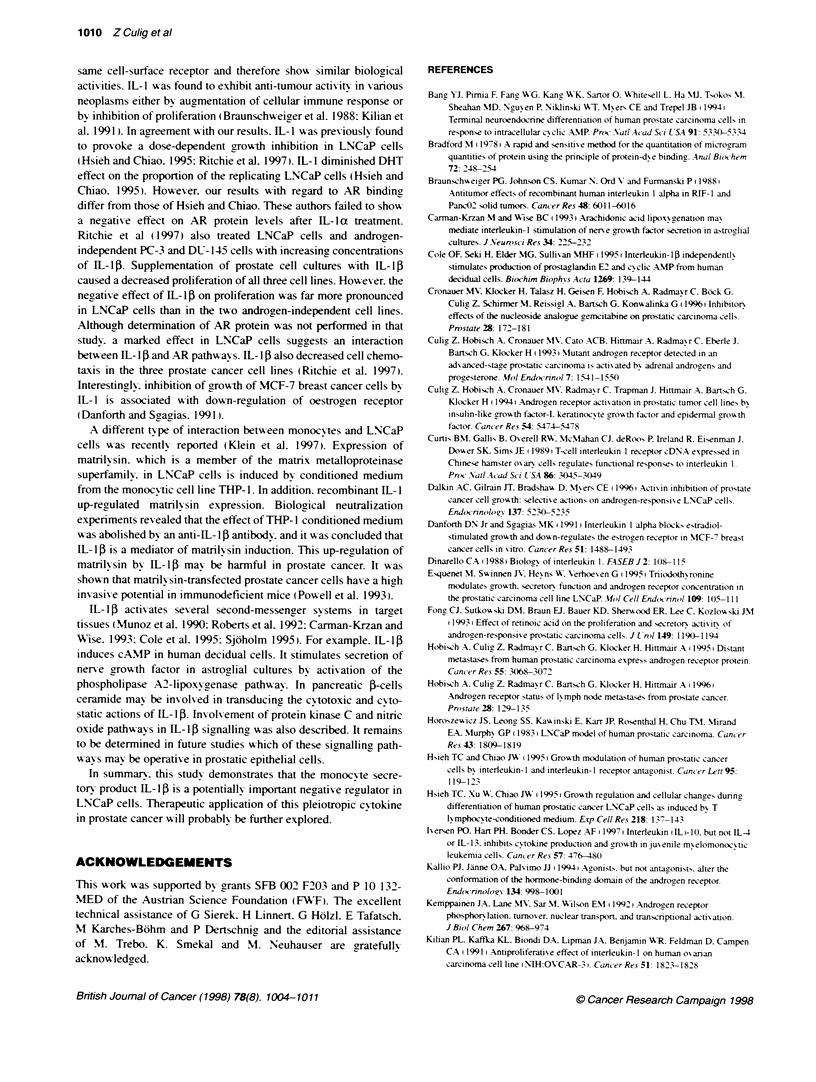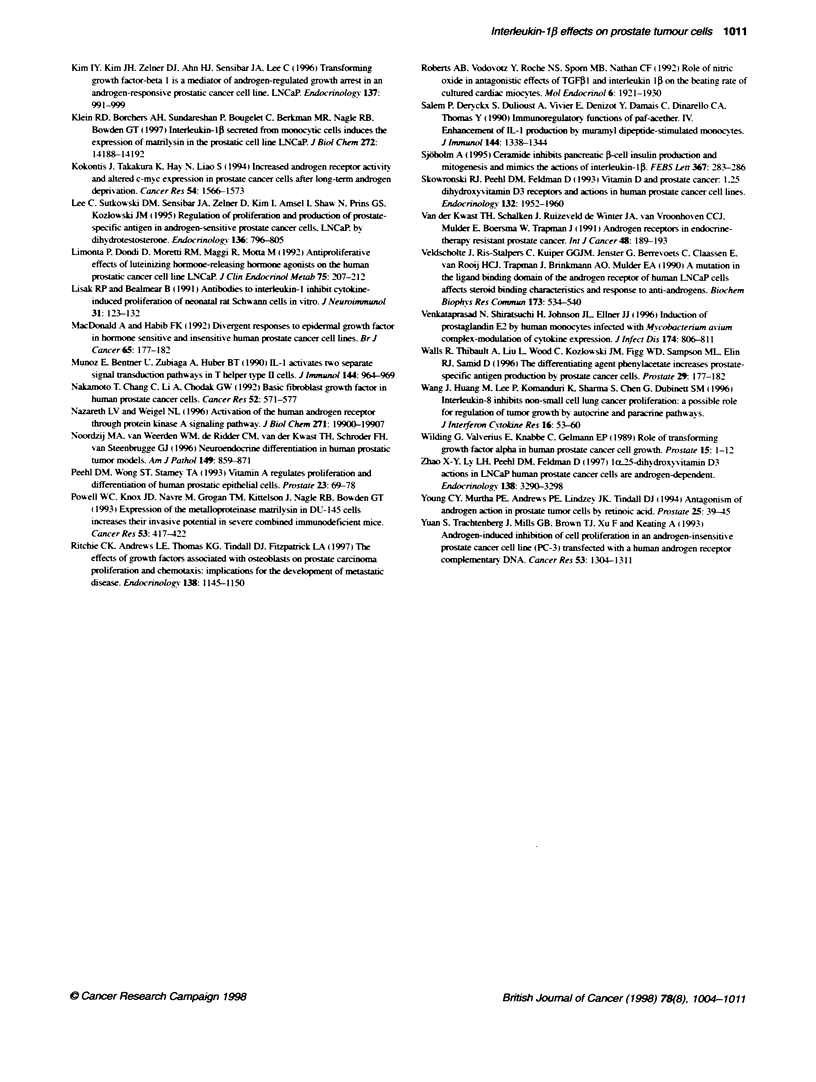# Interleukin 1beta mediates the modulatory effects of monocytes on LNCaP human prostate cancer cells.

**DOI:** 10.1038/bjc.1998.619

**Published:** 1998-10

**Authors:** Z. Culig, A. Hobisch, M. Herold, A. Hittmair, M. Thurnher, I. E. Eder, M. V. Cronauer, C. Rieser, R. Ramoner, G. Bartsch, H. Klocker, G. Konwalinka

**Affiliations:** Department of Urology, University of Innsbruck, Austria.

## Abstract

**Images:**


British Journal of Cancer (1998) 78(8), 1004–1011
© 1998 Cancer Research Campaign

### Interleukin 1β mediates the modulatory effects of monocytes on LNCaP human prostate cancer cells

Z Culig[1], A Hobisch[1], M Herold[2], A Hittmair[3], M Thurnher[1], IE Eder[1], MV Cronauer[1], C Rieser[1], R Ramoner[1], G Bartsch[1], H Klocker[1] and G Konwalinka[2]

[1]Departments of Urology, [2]Internal Medicine and [3]Pathology, University of Innsbruck, Anichstraße 35, A-6020 Innsbruck, Austria

**Summary** Proliferative and secretory responses in androgen-sensitive prostate cancer LNCaP cells are regulated by steroid and peptide hormones and by differentiation-promoting substances. In the present study, we evaluated whether peripheral blood monocytes that exhibit anti-tumour activity in haematopoietic and solid tumours influence growth and secretion in the LNCaP cell line. For this purpose, LNCaP cells were incubated with monocyte-conditioned medium (MCM), and proliferation as well as expression of androgen receptor (AR) and secretion of prostate-specific antigen (PSA) were assessed. Conditioned medium from monocytes reduced proliferation in a dose-dependent manner. Incubation with 40% MCM caused a 50% reduction in cell proliferation. AR protein decreased by 70% and PSA levels in supernatants from LNCaP cells were reduced by approximately 80% following treatment with MCM. We focused on the contribution of two major products of activated monocytes, prostaglandin $E_2$ and interleukin 1β (IL-1β), to the MCM modulatory action. LNCaP cells treated with prostaglandin $E_2$ showed neither a reduction in proliferation nor a down-regulation of AR and PSA levels. The effects of MCM on cellular proliferation, AR protein and PSA secretion were abolished by pretreatment of MCM with a neutralizing anti-IL-1β antibody. In addition, recombinant IL-1β was able to replace MCM for the inhibition of proliferation and down-regulation of AR and PSA proteins. LNCaP cells were shown to express the IL-1β receptor type I, which transduces IL-1β signal. Our findings reveal that monocyte-derived IL-1β inhibits the proliferation of androgen-responsive prostate tumour cells and reduces AR and PSA levels.

**Keywords:** prostate cancer cell; monocyte; proliferation; androgen receptor; prostate-specific antigen; interleukin 1β

The LNCaP cell line, which was derived from a lymph node metastasis of a patient who did not respond to endocrine therapy, is frequently used as a model for studying human prostate cancer (Horoszewicz et al, 1983). It is known that steroid and peptide hormones as well as differentiation-promoting substances regulate proliferative and secretory responses in LNCaP cells. The cells respond to androgen stimulation by accelerating division and producing prostate-specific antigen (PSA). The patterns of androgenic regulation of growth and secretory function in the LNCaP cell line are different (Lee et al, 1995). The maximal growth rate is achieved at 0.1 nM of dihydrotestosterone (DHT), whereas higher doses of this androgen induce growth arrest. It was proposed that the inhibition of cellular proliferation by high androgen doses is mediated by transforming growth factor β (TGF-β) (Kim et al, 1996). In contrast to the proliferative response, the percentage of PSA-positive cells and the levels of PSA protein in LNCaP supernatants increase at concentrations of 1 nM dihydrotestosterone and beyond (Lee et al, 1995). In addition to androgens, polypeptide growth factors such as epidermal growth factor (EGF), TGF-α, insulin-like growth factors and basic fibroblast growth factor (bFGF) are mitogenic for LNCaP cells (Wilding et al, 1989; MacDonald and Habib, 1992; Nakamoto et al, 1992; Ritchie et al, 1997). Positive effects of triiodothyronine and inhibitory effects of vitamin D, retinoic acid, luteinizing hormone-releasing hormone, phenylacetate and activin on growth of LNCaP cells have also been described (Limonta et al, 1992; Skowronski et al, 1993; Young et al, 1994; Esquenet et al, 1995; Dalkin et al, 1996; Walls et al, 1996). Secretion of PSA was found to be enhanced after treatment with triiodothyronine, vitamin D and phenylacetate (Skowronski et al, 1993; Esquenet et al, 1995; Walls et al, 1996), whereas divergent results on PSA regulation by retinoic acid were reported (Fong et al, 1993; Young et al, 1994). LNCaP cells express a mutant androgen receptor (AR), which binds oestrogenic and progestagenic steroids and non-steroidal anti-androgens hydroxyflutamide and nilutamide with higher affinity than the wild-type AR. These substances induce reporter gene activity in the presence of LNCaP AR more efficiently than in the presence of the wild-type AR (Veldscholte et al, 1990).

Studies on interactions between leucocytes that produce various cytokines and differentiation factors and prostate tumour cells may improve understanding of prostate cancer biology. In one of the initial studies in this field Hsieh et al (1995) have shown that phytohaemagglutinin (PHA)-stimulated lymphocytes produce substance(s) that reduce cell proliferation, increase expression of cytoskeleton proteins and down-regulate AR and PSA in LNCaP cells. These effects were observed after treatment with various concentrations of T-lymphocyte-conditioned media (TCM). However, the T-cell-derived factor(s) that mediate these effects have so far not been identified. Interactions between other cells of the immune system and various tumours have been described (Wang et al, 1996; Iversen et al, 1997). For example, monocytes were found to suppress cell viability and colony formation in human leukaemias and lung tumours. In this study, we address the issue of whether peripheral blood monocytes influence growth and secretion in androgen-responsive prostatic epithelial cells. We

*Received 13 November 1997*
*Revised 17 March 1998*
*Accepted 19 March 1998*

*Correspondence to:* Z Culig show that monocyte-derived interleukin 1β (IL-1β) is a mediator of the modulatory effects on LNCaP cells.

### MATERIALS AND METHODS

### Materials

MCDB-131 and RPMI-1640 media were purchased from Sigma (Deisenhofen, Germany) and HyClone (Logan, UT, USA) respectively. Fetal calf serum (FCS) and antibiotics (penicillin/streptomycin) were from Biological Industries (Kibutz Beth Haemek, Israel). Phosphate-buffered saline (PBS) was from PAA Laboratories (Linz, Austria). Cell culture vessels were from Costar (Cambridge, MA, USA), Sarstedt (Nümbrecht, Germany) and Falcon (Lincoln, NE, USA).

Phytohaemagglutinin-M (PHA-M) was purchased from Difco Laboratories (Detroit, MA, USA). The murine monoclonal anti-cytokeratin 8 and 18 antibody CAM 5.2 was purchased from Becton Dickinson (San Jose, CA, USA) and a polyclonal rabbit anti-IL-1β antibody from Genzyme (Cambridge, MA, USA). The mouse monoclonal antibody MEM18 (IgG1 anti-CD14) was from An Der Grub, Vienna, Austria. Biotinylated monoclonal antibody 6B5 (IgG2a anti-IL-1 receptor type I) was purchased from PharMingen (San Diego, CA, USA). Both radiolabelled (specific activity 83.2 Ci $mmol^{-1}$) and unlabelled synthetic androgen methyltrienolone (R 1881) were purchased from New England Nuclear (Dreieichenhain, Germany). IL-1β was provided by Sigma. Prostaglandin $E_2$ was from Boehringer Ingelheim (Heidelberg, Germany). The scintillation liquid Optiphase was from Pharmacia (Uppsala, Sweden). The commercial methylthiotetrazole (MTT) assay $E_4ZU$ was purchased from Biomedica (Vienna, Austria). An immunoenzymetric assay for the quantitative measurement of human IL-1β (minimum detectable concentration 2 pg $ml^{-1}$) was provided by Medgenix Diagnostics (Fleurus, Belgium). The PSA IMx enzyme immunoassay (sensitivity 0.1 ng $ml^{-1}$) was from Abbott Laboratories (Abbott Park, IL, USA). FACS Calibur apparatus and CellQuest software were from Becton-Dickinson.

### Culture of peripheral blood monocytes

Peripheral blood was collected from three healthy volunteers on several occasions. Monocytes were obtained from peripheral blood mononuclear cells after Ficoll separation by standard adherence (90 min, 37°C) and cultured in RPMI-1640 medium that was supplemented with 10% charcoal-stripped FCS (CS FCS). The purity of monocytes as determined by staining for naphthol acetate esterase and by measuring CD14 expression was > 90%. For preparation of monocyte-conditioned medium (MCM), $2.5 \times 10^5$ monocytes $ml^{-1}$ were cultured with and without PHA. After 48 h, the supernatants were cleared by centrifugation and frozen at − 20°C until use.

### Proliferation assays in LNCaP cells

The LNCaP cell line was purchased at passage 21 from the American Type Culture Collection (Bethesda, MD, USA). LNCaP cells were seeded into 24-well plates at $2 \times 10^4$ cells per well in MCDB-131 medium supplemented with 10% FCS. Medium was changed 24 h later and the final concentration of CS FCS was 3%. Culture medium was supplemented with MCM (10–60%), prostaglandin $E_2$ (1–10 μM) or IL-1β (0.1–10 ng $ml^{-1}$). Control experiments were carried out in the absence of any supplement in medium or in the presence of PHA-M. In neutralization experiments, MCM was preincubated with the antibody against IL-1β overnight. It has previously been determined that 1 mg of this antibody is capable of neutralizing approximately 1000 units of natural or recombinant IL-1β. Cell proliferation was determined after 72 h incubation by means of the MTT assay. This assay is based on the ability of living cells to reduce slightly coloured tetrazolium salts to intensely coloured formazan derivatives. The assay was performed as described previously (Cronauer et al, 1996). In selected experiments the MTT assay results were compared with those obtained with a cell counter and were found to be identical.

### Immunohistochemical analyses

Following treatment with MCM or medium without supplements, LNCaP cells were trypsinized, cytospun, resuspended in PBS, fixed in 1% paraformaldehyde, permeabilized by adding 0.1% Triton X-100 and stained for cyokeratin expression. Cytokeratin immunohistochemistry was performed according to a streptavidin–biotin–peroxidase protocol. The immunohistochemical procedure was described previously (Hobisch et al, 1995).

### Androgen receptor-binding assay

LNCaP cells were cultured in the absence (untreated control) or presence of respective supplements in 5% CS FCS for 72 h. Then they were scraped off, washed once, resuspended in medium and incubated with [$^3$H]methyltrienolone, at concentrations of 0.3–5 nM, for 90 min at room temperature. Non-specific binding was measured in the presence of a 200-fold molar excess of unlabelled methyltrienolone. The pellets were recovered after incubation by centrifugation (3800 ***g***, 3 min) and washed twice with 500 μl of ice-cold medium. The cell pellets were then lysed in 1 ml of scintillation liquid and the radioactivity was determined in a β-counter. Cellular protein was determined according to the method described by Bradford (1978). $B_{max}$ and $K_d$ were calculated by Scatchard analysis.

### Determination of prostate-specific antigen in supernatants from LNCaP cells

The cells were grown on 24-well plates in the presence of 5% CS FCS with or without supplements. The medium was removed after 72 h and the PSA level was measured by an enzyme immunoassay. PSA values were corrected for cell number according to the results of the proliferation assay.

### Determination of IL-1β concentration in monocyte conditioned media

IL-1β in MCM was determined by a solid-phase enzyme amplified sensitivity immunoassay. The assay is based on an oligoclonal system in which several monoclonal antibodies directed against distinct epitopes of IL-1β are used. The use of several distinct monoclonal antibodies avoids assay hyperspecificity. The assay was performed on a microtitre plate. Samples containing IL-1β react with capture antibodies coated on a plastic well and with monoclonal antibodies labelled with horseradish peroxidase. After

© Cancer Research Campaign 1998

Figure 1 Phenotypic characterization of peripheral blood monocytes and LNCaP cells. Monocytes (A) and LNCaP cells (B) were labelled with monoclonal antibodies specific for the antigen indicated followed by FITC-conjugated secondary antibody, and surface expression (bold lines) was determined with a flow cytometer as described in Materials and methods. Isotype-matched antibodies were included as controls (dotted lines). Fluorescence intensity (x-axis) is plotted against the number of cells (y-axis)

Figure 2 Morphology of untreated (A) and MCM-treated (B) LNCaP cells. MCM-treated cells are elongated and exhibit dendrite-like processes, which are indicated by arrows (original magnification × 100)

an incubation period of 2 h at room temperature the microtitre plate was washed three times to remove unbound enzyme-labelled antibodies. The revelation solution (tetramethylbenzydine-hydrogen peroxide) was added and incubated for 15 min at room temperature. The reaction was stopped with sulphuric acid and the microtitre plate was read at 450 nm. The concentrations of IL-1β in MCM ranged between 1.6 and 2.4 ng ml$^{-1}$.

### Measurement of surface antigen expression by flow cytometry

To determine surface antigen expression, peripheral blood monocytes were labelled with primary mouse monoclonal anti-CD14 antibody MEM18 followed by fluorescein isothiocyanate (FITC)-conjugated goat anti-mouse Ig. LNCaP cells were labelled with biotinylated primary mouse monoclonal anti-IL-1 receptor antibody 6B5 followed by FITC-conjugated streptavidin. Washes were in PBS containing 0.2% bovine serum albumin (BSA). After the last wash, the cells were stored in PBS containing 0.2% BSA and 2% formaldehyde. The samples were analysed on a FACS Calibur. Data were analysed and presented using CellQuest software.

### RESULTS

### Monocyte-conditioned medium-treated LNCaP cells show changes in cell shape

To study the influence of monocytes on prostate cancer cells, LNCaP cells were exposed to medium conditioned by peripheral blood monocytes homogenously positive for CD14 (Figure 1A). Distinct morphological changes in LNCaP cells were observed after treatment with monocyte-conditioned medium (MCM). The cells became elongated and showed dendrite-like processes, which were connected to each other (Figure 2). These morphological changes were notable even with 10% MCM in LNCaP culture medium. Expression of cytoskeletal proteins was evaluated by semiquantitative immunohistochemistry. A monoclonal antibody directed against luminal cytokeratins 8 and 18 was used for this purpose. The expression and staining intensity of these cytokeratins were previously reported to increase in the more differentiated prostatic luminal epithelium (Peehl et al, 1993). In the case of LNCaP cells treated with MCM, we observed neither an increase in the percentage of cytokeratin-immunopositive cells nor a change in staining intensity.

© Cancer Research Campaign 1998

**Figure 3** The effects of increasing concentrations of MCM on proliferation of LNCaP cells. The cells were incubated with MCM for 72 h and cell proliferation was assessed by the colorimetric MTT assay. Four measurements were performed. The results are expressed in per cent of untreated cells, which were set at 100%. Mean values and standard deviations were calculated. *$P < 0.05$, MCM treatment vs untreated cells (0) and R1881 + MCM vs R1881, Mann–Whitney *U*-test

**Figure 4** Methyltrienolone binding in untreated (control) and MCM-treated LNCaP cells. After 72 h of MCM treatment, LNCaP cells were incubated for 90 min with increasing concentrations of [$^3$H]-methyltrienolone. Non-specific binding was measured in the presence of a 200-fold molar excess of unlabelled methyltrienolone. The cells were washed twice with ice-cold medium and specific binding was determined. ○, Control, $K_d$ = 1.1 nmol, $B_{max}$ = 0.60 fmol μg$^{-1}$ protein; □, 40% MCM, $K_d$ = 1.0 nmol, $B_{max}$ = 0.16 fmol μg$^{-1}$ protein

### Monocyte-conditioned medium causes a dose-dependent reduction in cellular proliferation

LNCaP cells were cultured in the absence or presence of MCM for 3 days. After this period, cellular growth was assessed by means of the MTT assay. Conditioned medium from PHA-M-activated monocytes reduced cellular proliferation in a dose-dependent manner (Figure 3). MCM at 40% reduced proliferation by 50%. Higher concentrations of MCM in culture media did not result in any further reduction in cell number. PHA-M itself did not display any effect on the proliferation of LNCaP cells. Conditioned medium from monocytes that were not treated with PHA-M induced a similar decrease in cell proliferation (data not shown). Thus, it seems that the presence of PHA in monocyte cultures is not required for inhibition of growth of LNCaP cells. The proliferative effect of 0.01 nM of R1881 was abolished by 40% MCM (Figure 3).

### Androgen receptor levels and PSA secretion decrease after treatment with MCM

Specific binding of radioactively labelled methyltrienolone in MCM-treated LNCaP cells as well as in controls was determined. MCM caused a dose-dependent reduction in androgen binding. Figure 4 shows a decrease in specific binding of [$^3$H]R1881 in cells treated with 40% MCM. The maximum decrease in the AR level was approximately 70% in the presence of 40% MCM. There was no change in AR binding affinity following MCM treatment. Secretion of the AR-regulated PSA protein was measured in supernatants from LNCaP cells after 72 h of incubation with MCM. A decrease in PSA was observed in the presence of 20–40% of MCM in culture media. PSA protein was reduced by 80% with 40% MCM. MCM also antagonized stimulatory effects of the synthetic androgen R1881 on PSA secretion into the supernatant (Figure 5).

### IL-1β is responsible for the modulatory effects of MCM

We focused on the contribution of individual factors to the MCM modulatory effects. Initially, we investigated the role of prostaglandin $E_2$, which is a major product of activated monocytes (Venkataprasad et al, 1996). LNCaP cells treated with prostaglandin $E_2$ showed neither a reduction in proliferation nor a down-regulation of AR and PSA levels. Thus, prostaglandin $E_2$ can be excluded as a mediator of the inhibitory effects of monocytes on androgen-responsive prostate cancer cells. Next, we examined the role of the proinflammatory cytokine IL-1β, which exhibits anti-tumour activity in several models (Kilian et al, 1991; Braunschweiger et al, 1996), in the interaction between the immune system and prostate cancer cells. In order to determine whether IL-1β mediates MCM effects on LNCaP cells, MCM was preincubated with a neutralizing polyclonal anti-IL-1β antibody. The effectiveness of this antibody was previously demonstrated in other assay systems (Salem et al, 1990; Lisak and Bealmear, 1991). MCM pretreated with the antibody did not reduce LNCaP cell proliferation (Figure 6A). The MCM effect on AR protein was completely abolished after preincubation of MCM with the polyclonal anti-IL-1β antibody (Figure 6B), and the PSA levels were almost completely restored (Figure 6C). Moreover, addition of recombinant IL-1β to cultures of LNCaP cells exerted effects that

© Cancer Research Campaign 1998 

**Figure 5** Regulation of PSA secretion in LNCaP cells by MCM. The cells were incubated in the absence or presence of MCM for 72 h. PSA levels were measured by an enzyme immunoassay and normalized according to cell proliferation as assessed by means of the MTT assay. Data are expressed as nanograms of PSA per unit of absorbance at 450 nm (UA). Mean values and standard deviations were calculated from four independent experiments. *$P < 0.05$. MCM treatment vs untreated cells (0) and R1881 + MCM vs R1881. Mann–Whitney *U*-test were almost identical to those of MCM. Cell proliferation was reduced dose dependently (Figure 6A), the AR protein levels decreased (Figure 6D) and PSA secretion was diminished (Figure 6C). Finally, LNCaP cells were shown to express the IL-1β receptor type I (Figure 1B), which transduces IL-1β signal (Curtis et al, 1989).

### DISCUSSION

The major finding of the present study is that peripheral blood monocytes are capable of modulating cellular events in androgen-responsive LNCaP cells by secreting IL-1β. This ability of monocytes is similar to that previously reported for T lymphocytes in the same cell line (Hsieh et al, 1995). Both cell types secrete substances that cause a reduction in cellular proliferation, diminish levels of AR protein and down-regulate PSA in LNCaP cells. In our experiments, MCM was effective regardless of the presence of PHA in monocyte cultures. In contrast, unstimulated lymphocytes provoked only a minimal growth inhibition of LNCaP cells (Hsieh et al, 1995).

LNCaP cells became elongated and exhibited dendrite-like processes after contact with MCM and TCM. Similar changes in cell shape were described after treatment of LNCaP cells with analogues of cAMP (Bang et al, 1994). There was, however, one difference between MCM- and TCM-treated LNCaP cells. The expression of cytokeratins 8 and 18 and their staining intensity increased twofold after incubation with TCM as determined by a total intensity score procedure (Hsieh et al, 1995). In the case of MCM we did not see any differences in the expression of these cytoskeleton proteins by semiquantitative immunohistochemistry. Contrasting results on expression of cytokeratins may be due to use of different antibodies and immunohistochemical techniques. It was suggested by others that TCM- and cAMP analogue-treated LNCaP cells undergo neuroendocrine differentiation (Bang et al, 1994; Hsieh et al, 1995). TCM treatment provoked an increase in total intensity score of two neuroendocrine markers, neuron-specific enolase and serotonin. However, the concept of neuroendocrine differentiation of this cell line was questioned recently (Noordzij et al, 1996). In that publication and in our previous experimental studies neuroendocrine cells within the LNCaP cell line were not identified (unpublished data). Thus, a differentiation process induced by leucocytes in androgen-responsive prostate cancer cells is not well characterized to date.

The MCM-induced growth-inhibitory effect was associated with decreased AR expression. Association between AR expression and growth regulation of prostate cancer cells can be studied in AR-positive LNCaP cells and in AR cDNA-transfected PC-3 cells. It was demonstrated that the expression of AR in PC-3 cells leads to a decrease in proliferation (Yuan et al, 1993). We infer that more reliable information on AR involvement in growth regulation may be obtained in its natural cellular environment, i.e. in LNCaP cells. Growth inhibition and AR reduction were previously observed in LNCaP cells incubated with retinoic acid and TCM (Young et al, 1994; Hsieh et al, 1995). Regarding AR expression, conditioned media from T lymphocytes and monocytes, and retinoic acid differ from anti-androgens cyproterone acetate, hydroxyflutamide and bicalutamide, which are commonly used in endocrine therapy for prostate cancer. These compounds bind to the AR with low affinity and do not permit acquisition of a transcriptionally active form of the receptor (Kemppainen et al, 1992; Kallio et al, 1994). However, they did not down-regulate AR protein itself. AR is expressed in relapsed prostate tumours and in their metastases, which were obtained before onset of therapy and during tumour progression (van der Kwast et al, 1991; Hobisch et al, 1995, 1996). Several recent publications support the view that reduction in androgen concentration is not sufficient to prevent transmission of extracellular signals via the AR (Veldscholte et al, 1990; Culig et al, 1993, 1994; Kokontis et al, 1994; Nazareth and Weigel, 1996). Prostate cancer cells may adapt to an environment with very low androgen concentration by increasing their AR expression and transcriptional activity (Kokontis et al, 1994). Furthermore, mutant ARs discovered in prostate cancer frequently exhibit a gain in function: they are efficiently activated by other steroids and non-steroidal AR antagonists (Veldscholte et al, 1990; Culig et al, 1993). Finally, AR activity is up-regulated by several non-steroidal substances such as polypeptide growth factors and second messengers (Culig et al, 1994; Nazareth and Weigel, 1996). Therefore, strategies aimed at reducing AR levels, such as administration of secretory products from the immune cells, may be beneficial in metastatic prostate cancer. AR down-regulation was followed by reduction of PSA protein in LNCaP supernatants. Thus, the effect of MCM is different to that of phenylacetate and vitamin D, substances that have been reported to inhibit proliferation of LNCaP cells and increase PSA secretion (Walls et al, 1996; Zhao et al, 1997).

The mediator role of IL-1β was revealed in experiments in which the neutralizing anti-IL-1β antibody abolished the effects of MCM. Furthermore, IL-1β itself exhibited a dose-dependent inhibition on LNCaP proliferation, AR protein level and PSA secretion. In contrast to our findings with MCM, a compound that is responsible

© Cancer Research Campaign 1998

**Figure 6** The effects of pretreatment of MCM with the neutralizing polyclonal anti-IL-1β antibody and the effects of IL-1β on (**A**) proliferation. (**B**. **D**) cellular AR protein levels and (**C**) PSA protein in supernatants of LNCaP cells. Four independent experiments were performed. (AB. antibody): **A**. **C** **P* < 0.05. IL-1β treatment vs untreated cells (0) and antibody pretreatment vs treatment without antibody. Mann–Whitney *U*-test. **B**. **D**: ○. Control. $K_d$ = 1.0 nmol. $B_{max}$ = 0.62 fmol μg⁻¹ protein: □. 40% MCM. $K_d$ = 1.6 nmol. $B_{max}$ = 0.19 fmol μg⁻¹ protein: ★. 40% MCM + AB. $K_d$ = 1.2 nmol. $B_{max}$ = 0.60 fmol μg⁻¹ protein for the growth-modulatory effects of TCM on androgen-responsive prostate cancer cells has not been identified. Antibodies against TNF-α. TGF-β. FGF. EGF and IL-2. -4. -5. -6 or -8 were tested. but none of them was capable of neutralizing TCM effects (Hsieh et al. 1995). Based on semipurification of growth-modulatory activity. it was proposed that a protein in the molecular weight range of 13–24 kDa is responsible for TCM action. It is not unlikely that growth-modulatory effects of TCM and MCM could be. at least in part. attributed to the same cytokine.

Monocytes are known as the major source of secreted IL-1 (Dinarello. 1988). Human IL-1β is a 17-kDa protein that is secreted during an inflammatory process. Two functionally almost equivalent forms of IL-1. IL-1α and IL-1β. which display about 27% homology at the protein level. exist. Both forms of IL-1 bind to the same cell-surface receptor and therefore show similar biological activities. IL-1 was found to exhibit anti-tumour activity in various neoplasms either by augmentation of cellular immune response or by inhibition of proliferation (Braunschweiger et al. 1988; Kilian et al. 1991). In agreement with our results, IL-1 was previously found to provoke a dose-dependent growth inhibition in LNCaP cells (Hsieh and Chiao, 1995; Ritchie et al. 1997). IL-1 diminished DHT effect on the proportion of the replicating LNCaP cells (Hsieh and Chiao, 1995). However, our results with regard to AR binding differ from those of Hsieh and Chiao. These authors failed to show a negative effect on AR protein levels after IL-1α treatment. Ritchie et al (1997) also treated LNCaP cells and androgen-independent PC-3 and DU-145 cells with increasing concentrations of IL-1β. Supplementation of prostate cell cultures with IL-1β caused a decreased proliferation of all three cell lines. However, the negative effect of IL-1β on proliferation was far more pronounced in LNCaP cells than in the two androgen-independent cell lines. Although determination of AR protein was not performed in that study, a marked effect in LNCaP cells suggests an interaction between IL-1β and AR pathways. IL-1β also decreased cell chemotaxis in the three prostate cancer cell lines (Ritchie et al. 1997). Interestingly, inhibition of growth of MCF-7 breast cancer cells by IL-1 is associated with down-regulation of oestrogen receptor (Danforth and Sgagias, 1991).

A different type of interaction between monocytes and LNCaP cells was recently reported (Klein et al. 1997). Expression of matrilysin, which is a member of the matrix metalloproteinase superfamily, in LNCaP cells is induced by conditioned medium from the monocytic cell line THP-1. In addition, recombinant IL-1 up-regulated matrilysin expression. Biological neutralization experiments revealed that the effect of THP-1 conditioned medium was abolished by an anti-IL-1β antibody, and it was concluded that IL-1β is a mediator of matrilysin induction. This up-regulation of matrilysin by IL-1β may be harmful in prostate cancer. It was shown that matrilysin-transfected prostate cancer cells have a high invasive potential in immunodeficient mice (Powell et al. 1993).

IL-1β activates several second-messenger systems in target tissues (Munoz et al. 1990; Roberts et al. 1992; Carman-Krzan and Wise, 1993; Cole et al. 1995; Sjöholm 1995). For example, IL-1β induces cAMP in human decidual cells. It stimulates secretion of nerve growth factor in astroglial cultures by activation of the phospholipase A2-lipoxygenase pathway. In pancreatic β-cells ceramide may be involved in transducing the cytotoxic and cytostatic actions of IL-1β. Involvement of protein kinase C and nitric oxide pathways in IL-1β signalling was also described. It remains to be determined in future studies which of these signalling pathways may be operative in prostatic epithelial cells.

In summary, this study demonstrates that the monocyte secretory product IL-1β is a potentially important negative regulator in LNCaP cells. Therapeutic application of this pleiotropic cytokine in prostate cancer will probably be further explored.

### ACKNOWLEDGEMENTS

This work was supported by grants SFB 002 F203 and P 10 132-MED of the Austrian Science Foundation (FWF). The excellent technical assistance of G Sierek, H Linnert, G Hölzl, E Tafatsch, M Karches-Böhm and P Dertschnig and the editorial assistance of M. Trebo, K. Smekal and M. Neuhauser are gratefully acknowledged.

### REFERENCES

Bang YJ, Pirnia F, Fang WG, Kang WK, Sartor O, Whitesell L, Ha MJ, Tsokos M, Sheahan MD, Nguyen P, Niklinski WT, Myers CE and Trepel JB (1994) Terminal neuroendocrine differentiation of human prostate carcinoma cells in response to intracellular cyclic AMP. *Proc Natl Acad Sci USA* **91**: 5330–5334

Bradford M (1978) A rapid and sensitive method for the quantitation of microgram quantities of protein using the principle of protein-dye binding. *Anal Biochem* **72**: 248–254

Braunschweiger PG, Johnson CS, Kumar N, Ord V and Furmanski P (1988) Antitumor effects of recombinant human interleukin 1 alpha in RIF-1 and Panc02 solid tumors. *Cancer Res* **48**: 6011–6016

Carman-Krzan M and Wise BC (1993) Arachidonic acid lipoxygenation may mediate interleukin-1 stimulation of nerve growth factor secretion in astroglial cultures. *J Neurosci Res* **34**: 225–232

Cole OF, Seki H, Elder MG, Sullivan MHF (1995) Interleukin-1β independently stimulates production of prostaglandin E2 and cyclic AMP from human decidual cells. *Biochim Biophys Acta* **1269**: 139–144

Cronauer MV, Klocker H, Talasz H, Geisen F, Hobisch A, Radmayr C, Böck G, Culig Z, Schirmer M, Reissigl A, Bartsch G, Konwalinka G (1996) Inhibitory effects of the nucleoside analogue gemcitabine on prostatic carcinoma cells. *Prostate* **28**: 172–181

Culig Z, Hobisch A, Cronauer MV, Cato ACB, Hittmair A, Radmayr C, Eberle J, Bartsch G, Klocker H (1993) Mutant androgen receptor detected in an advanced-stage prostatic carcinoma is activated by adrenal androgens and progesterone. *Mol Endocrinol* **7**: 1541–1550

Culig Z, Hobisch A, Cronauer MV, Radmayr C, Trapman J, Hittmair A, Bartsch G, Klocker H (1994) Androgen receptor activation in prostatic tumor cell lines by insulin-like growth factor-I, keratinocyte growth factor and epidermal growth factor. *Cancer Res* **54**: 5474–5478

Curtis BM, Gallis B, Overell RW, McMahan CJ, deRoos P, Ireland R, Eisenman J, Dower SK, Sims JE (1989) T-cell interleukin 1 receptor cDNA expressed in Chinese hamster ovary cells regulates functional responses to interleukin 1. *Proc Natl Acad Sci USA* **86**: 3045–3049

Dalkin AC, Gilrain JT, Bradshaw D, Myers CE (1996) Activin inhibition of prostate cancer cell growth: selective actions on androgen-responsive LNCaP cells. *Endocrinology* **137**: 5230–5235

Danforth DN Jr and Sgagias MK (1991) Interleukin 1 alpha blocks estradiol-stimulated growth and down-regulates the estrogen receptor in MCF-7 breast cancer cells in vitro. *Cancer Res* **51**: 1488–1493

Dinarello CA (1988) Biology of interleukin 1. *FASEB J* **2**: 108–115

Esquenet M, Swinnen JV, Heyns W, Verhoeven G (1995) Triiodothyronine modulates growth, secretory function and androgen receptor concentration in the prostatic carcinoma cell line LNCaP. *Mol Cell Endocrinol* **109**: 105–111

Fong CJ, Sutkowski DM, Braun EJ, Bauer KD, Sherwood ER, Lee C, Kozlowski JM (1993) Effect of retinoic acid on the proliferation and secretory activity of androgen-responsive prostatic carcinoma cells. *J Urol* **149**: 1190–1194

Hobisch A, Culig Z, Radmayr C, Bartsch G, Klocker H, Hittmair A (1995) Distant metastases from human prostatic carcinoma express androgen receptor protein. *Cancer Res* **55**: 3068–3072

Hobisch A, Culig Z, Radmayr C, Bartsch G, Klocker H, Hittmair A (1996) Androgen receptor status of lymph node metastases from prostate cancer. *Prostate* **28**: 129–135

Horoszewicz JS, Leong SS, Kawinski E, Karr JP, Rosenthal H, Chu TM, Mirand EA, Murphy GP (1983) LNCaP model of human prostatic carcinoma. *Cancer Res* **43**: 1809–1819

Hsieh TC and Chiao JW (1995) Growth modulation of human prostatic cancer cells by interleukin-1 and interleukin-1 receptor antagonist. *Cancer Lett* **95**: 119–123

Hsieh TC, Xu W, Chiao JW (1995) Growth regulation and cellular changes during differentiation of human prostatic cancer LNCaP cells as induced by T lymphocyte-conditioned medium. *Exp Cell Res* **218**: 137–143

Iversen PO, Hart PH, Bonder CS, Lopez AF (1997) Interleukin (IL)-10, but not IL-4 or IL-13, inhibits cytokine production and growth in juvenile myelomonocytic leukemia cells. *Cancer Res* **57**: 476–480

Kallio PJ, Jänne OA, Palvimo JJ (1994) Agonists, but not antagonists, alter the conformation of the hormone-binding domain of the androgen receptor. *Endocrinology* **134**: 998–1001

Kemppainen JA, Lane MV, Sar M, Wilson EM (1992) Androgen receptor phosphorylation, turnover, nuclear transport, and transcriptional activation. *J Biol Chem* **267**: 968–974

Kilian PL, Kaffka KL, Biondi DA, Lipman JA, Benjamin WR, Feldman D, Campen CA (1991) Antiproliferative effect of interleukin-1 on human ovarian carcinoma cell line (NIH:OVCAR-3). *Cancer Res* **51**: 1823–1828

© Cancer Research Campaign 1998

Kim IY. Kim JH. Zelner DJ. Ahn HJ. Sensibar JA. Lee C (1996) Transforming growth factor-beta 1 is a mediator of androgen-regulated growth arrest in an androgen-responsive prostatic cancer cell line. LNCaP. *Endocrinology* **137**: 991–999

Klein RD. Borchers AH. Sundareshan P. Bougelet C. Berkman MR. Nagle RB. Bowden GT (1997) Interleukin-1β secreted from monocytic cells induces the expression of matrilysin in the prostatic cell line LNCaP. *J Biol Chem* **272**: 14188–14192

Kokontis J. Takakura K. Hay N. Liao S (1994) Increased androgen receptor activity and altered c-myc expression in prostate cancer cells after long-term androgen deprivation. *Cancer Res* **54**: 1566–1573

Lee C. Sutkowski DM. Sensibar JA. Zelner D. Kim I. Amsel I. Shaw N. Prins GS. Kozlowski JM (1995) Regulation of proliferation and production of prostate-specific antigen in androgen-sensitive prostate cancer cells. LNCaP. by dihydrotestosterone. *Endocrinology* **136**: 796–805

Limonta P. Dondi D. Moretti RM. Maggi R. Motta M (1992) Antiproliferative effects of luteinizing hormone-releasing hormone agonists on the human prostatic cancer cell line LNCaP. *J Clin Endocrinol Metab* **75**: 207–212

Lisak RP and Bealmear B (1991) Antibodies to interleukin-1 inhibit cytokine-induced proliferation of neonatal rat Schwann cells in vitro. *J Neuroimmunol* **31**: 123–132

MacDonald A and Habib FK (1992) Divergent responses to epidermal growth factor in hormone sensitive and insensitive human prostate cancer cell lines. *Br J Cancer* **65**: 177–182

Munoz E. Bentner U. Zubiaga A. Huber BT (1990) IL-1 activates two separate signal transduction pathways in T helper type II cells. *J Immunol* **144**: 964–969

Nakamoto T. Chang C. Li A. Chodak GW (1992) Basic fibroblast growth factor in human prostate cancer cells. *Cancer Res* **52**: 571–577

Nazareth LV and Weigel NL (1996) Activation of the human androgen receptor through protein kinase A signaling pathway. *J Biol Chem* **271**: 19900–19907

Noordzij MA. van Weerden WM. de Ridder CM. van der Kwast TH. Schroder FH. van Steenbrugge GJ (1996) Neuroendocrine differentiation in human prostatic tumor models. *Am J Pathol* **149**: 859–871

Peehl DM. Wong ST. Stamey TA (1993) Vitamin A regulates proliferation and differentiation of human prostatic epithelial cells. *Prostate* **23**: 69–78

Powell WC. Knox JD. Navre M. Grogan TM. Kittelson J. Nagle RB. Bowden GT (1993) Expression of the metalloproteinase matrilysin in DU-145 cells increases their invasive potential in severe combined immunodeficient mice. *Cancer Res* **53**: 417–422

Ritchie CK. Andrews LE. Thomas KG. Tindall DJ. Fitzpatrick LA (1997) The effects of growth factors associated with osteoblasts on prostate carcinoma proliferation and chemotaxis: implications for the development of metastatic disease. *Endocrinology* **138**: 1145–1150

Roberts AB. Vodovotz Y. Roche NS. Sporn MB. Nathan CF (1992) Role of nitric oxide in antagonistic effects of TGFβ1 and interleukin 1β on the beating rate of cultured cardiac miocytes. *Mol Endocrinol* **6**: 1921–1930

Salem P. Deryckx S. Dulioust A. Vivier E. Denizot Y. Damais C. Dinarello CA. Thomas Y (1990) Immunoregulatory functions of paf-acether. IV. Enhancement of IL-1 production by muramyl dipeptide-stimulated monocytes. *J Immunol* **144**: 1338–1344

Sjöholm A (1995) Ceramide inhibits pancreatic β-cell insulin production and mitogenesis and mimics the actions of interleukin-1β. *FEBS Lett* **367**: 283–286

Skowronski RJ. Peehl DM. Feldman D (1993) Vitamin D and prostate cancer: 1.25 dihydroxyvitamin D3 receptors and actions in human prostate cancer cell lines. *Endocrinology* **132**: 1952–1960

Van der Kwast TH. Schalken J. Ruizeveld de Winter JA. van Vroonhoven CCJ. Mulder E. Boersma W. Trapman J (1991) Androgen receptors in endocrine-therapy resistant prostate cancer. *Int J Cancer* **48**: 189–193

Veldscholte J. Ris-Stalpers C. Kuiper GGJM. Jenster G. Berrevoets C. Claassen E. van Rooij HCJ. Trapman J. Brinkmann AO. Mulder EA (1990) A mutation in the ligand binding domain of the androgen receptor of human LNCaP cells affects steroid binding characteristics and response to anti-androgens. *Biochem Biophys Res Commun* **173**: 534–540

Venkataprasad N. Shiratsuchi H. Johnson JL. Ellner JJ (1996) Induction of prostaglandin E2 by human monocytes infected with *Mycobacterium avium* complex-modulation of cytokine expression. *J Infect Dis* **174**: 806–811

Walls R. Thibault A. Liu L. Wood C. Kozlowski JM. Figg WD. Sampson ML. Elin RJ. Samid D (1996) The differentiating agent phenylacetate increases prostate-specific antigen production by prostate cancer cells. *Prostate* **29**: 177–182

Wang J. Huang M. Lee P. Komanduri K. Sharma S. Chen G. Dubinett SM (1996) Interleukin-8 inhibits non-small cell lung cancer proliferation: a possible role for regulation of tumor growth by autocrine and paracrine pathways. *J Interferon Cytokine Res* **16**: 53–60

Wilding G. Valverius E. Knabbe C. Gelmann EP (1989) Role of transforming growth factor alpha in human prostate cancer cell growth. *Prostate* **15**: 1–12

Zhao X-Y. Ly LH. Peehl DM. Feldman D (1997) 1α,25-dihydroxyvitamin D3 actions in LNCaP human prostate cancer cells are androgen-dependent. *Endocrinology* **138**: 3290–3298

Young CY. Murtha PE. Andrews PE. Lindzey JK. Tindall DJ (1994) Antagonism of androgen action in prostate tumor cells by retinoic acid. *Prostate* **25**: 39–45

Yuan S. Trachtenberg J. Mills GB. Brown TJ. Xu F and Keating A (1993) Androgen-induced inhibition of cell proliferation in an androgen-insensitive prostate cancer cell line (PC-3) transfected with a human androgen receptor complementary DNA. *Cancer Res* **53**: 1304–1311

© Cancer Research Campaign 1998